# Early Complication in Sickle Cell Anemia Children due to *A(TA)_n_TAA* Polymorphism at the Promoter of *UGT1A1* Gene

**DOI:** 10.1155/2013/173474

**Published:** 2013-07-28

**Authors:** Leila Chaouch, Emna Talbi, Imen Moumni, Arij Ben Chaabene, Miniar Kalai, Dorra Chaouachi, Fethi Mallouli, Abderraouf Ghanem, Salem Abbes

**Affiliations:** ^1^Laboratoire d'Hématologie Moléculaire et Cellulaire, Institut Pasteur de Tunis, Université de Tunis El Manar, Tunis, Tunisia; ^2^Université de Tunis El Manar, Tunis, Tunisia; ^3^Département de Biologie Clinique, Institut Salah Azaiez de Cancer, Université de Tunis El Manar, Tunis, Tunisia; ^4^Département de Biochimie, Hôpital de Traumatologie et des Grands Brulés, Université de Tunis El Manar, Ben Arous, Tunisia

## Abstract

*Aim*. To determine the implication of the polymorphism, namely, *A(TA)_n_TAA* of *UGT1A1* in lithogenesis for the first time in Tunisia among sickle cell anemia (SCA) children patients. *Material and Methods*. Our study was performed in 2010 and it involved 76 subjects chosen as control group characterized with normal hemoglobin status and presence of cholelithiasis and 102 SCA pediatric patients among whom 52 have cholelithiasis. We analyzed the polymorphism *A(TA)_n_TAA* at the *UGT1A1* promoter and the relationships between the various *A(TA)_n_TAA* genotypes and alleles and bilirubin levels and occurrence of cholelithiasis. *Results and Discussion*. The repartition of genotypes found according to serum bilirubin level shows a significant association between genotypes carrying variant (TA)_7_ and hyperbilirubinemia (*P* < 0.05). We demonstrated the association of two genotypes with gallstones formation among SCA children patients: (TA)_7_/(TA)_7_ and (TA)_7_/(TA)_8_ with *P* = 8.1 × 10^−8^ and *P* = 0.01, respectively. (TA)_7_ and (TA)_8_ allele variants act as a risk factor for early gallstones formation in SCA patients with *P* = 5.8 × 10^−9^ and *P* = 0.01, respectively. As for the control group only the genotype (TA)_7_/(TA)_7_ presented a risk factor for gallstones formation. *Conclusion*. The novelty of this report is that it is the first time that a similar study was made on the Tunisian children sickle cell population and that the results show a clear association of (TA)_7_ variant in early gallstones formation in Tunisian SCA children. Interestingly our findings highlighted the association of (TA)_8_ variant as well, which was not found in previous studies.

## 1. Introduction

SCA is a heterogeneous monogenic disease due to a single mutation A/T at the sixth codon of the *β*-globin gene (*β*S) [1]. The clinical complications arising from sickle cell disease include vasoo-cclusive crisis and its outcomes [1]. As a result of chronic hemolysis, hyperbilirubinemia is often observed leading to the formation of pigment cholelithiasis which could be busted by the presence of *UGT1A1 *defects. Indeed *UGT1A1* gene encodes the uridine diphosphate glucuronosyltransferase 1A1, enzyme responsible for bilirubin glucuronidation [2]. The *UGT1A1* gene is located in chromosome 2q37 [3]. Various *UGT1A1* gene defects and polymorphisms have been described so far at the origin of reduced enzyme activity [4]. Among these, a variation in the number of TA repeat at the *A(TA)*_6_ *TAA* nucleotide sequence in the promoter region, considered as the wild type. In fact, the addition of an extra (TA) at this sequence leads to a variant *A(TA)*_7_ *TAA *which was described to cause reduced glucuronidation and hyperbilirubinemia associated with the Gilbert syndrome [2, 5]. This variation at the promoter seems to interfere with binding of the transcription factor IID which is responsible for the transcription of *UGT1A1* gene. In fact, The *A(TA)*_n_ *TAA* element is the binding site for transcription factor IID, which is one of the factors responsible for the initiation of transcription and the presence of this longer *A(TA)*_n_ *TAA* element in the promoter region of the gene for bilirubin UDP-glucuronosyltransferase 1 resulting in reduced expression of bilirubin-UGT1 (30% of normal) and hence causing unconjugated hyperbilirubinemia [3]. Studies of a possible association between polymorphisms of candidate genes related to the modulation of clinical complications of SCA have shown that sickle cell patients who carry the variation (TA)_7_ are favorable for gallstone formation [4–11]. Besides, other studies have shown the correlation of cholelithiasis and *A(TA)*_7_ *TAA* variant of *UGT1A1* promoter with chronic hemolytic diseases such as thalassemia minor, which represent a risk factor for cholelithiasis and the Gilbert mutation further increases this risk [12–16]. The prevalence of cholelithiasis observed in SCA children is about 30% reported for different ethnical groups (United States, Guadeloupe) [17, 18]. 

In this paper, we intend to study the impact of *A(TA)*_n_ *TAA *variation at the *UGT1A1* gene promoter on hyperbilirubinemia and on the occurrence of cholelithiasis for the first time among SCA Tunisian children. SCA is the second sickle cell hemoglobinopathy after *β*-thalassemia in Tunisia, representing a real public health problem. The average frequency of the trait in our country is 1.89% [19]. The organization of care of sickle cell disease children in Tunisia is the National Center of Bone Marrow Transplantation.

## 2. Methods 

### 2.1. Subjects

76 subjects with cholelithiasis and 102 sickle cell patients were involved in this study performed in 2010. Patients were selected on the basis of homozygosity for *β*-globin gene from National Center of Bone Marrow Transplantation, Tunis, Tunisia. All SCA patients are children (less than 16 years old) and were characterized by hyperbilirubinemia and 52 of them have cholelithiasis.

### 2.2. Methods

#### 2.2.1. Clinical Events Analyzed

Liver/biliary ultrasound scans were performed annually to detect cholelithiasis only in SCA patients over the age of three years. Cholelithiasis was diagnosed for all patients on the basis of echodense images within the gallbladder with acoustic shadowing or gravitational change in position.

#### 2.2.2. Laboratory Methods

Diagnosis of sickle cell patient is performed using cation-exchange high-performance liquid chromatography (HPLC) (D10 Biorad) and further confirmation by means of molecular diagnosis by restriction fragment length polymorphism (RFLP) using DdeI as previously described by Bachir 2000 [20]. Biochemical data were averaged for each patient in steady state (at least three values). We determined total and fetal hemoglobin (HbF) concentrations (D10, Biorad) and reticulocyte count and other hematologic parameters using (ABX pentra 60c+). Total unconjugated and conjugated bilirubin concentrations in serum were determined by a standardized colorimetric procedure (Cobras Integra, Meylan, France).

#### 2.2.3. *A(TA)*_n_ *TAA* Genotyping

Genomic DNA was isolated from white blood cells of total blood using standard method (phenol/chloroform). *A(TA)*_n_ *TAA* sequences were genotyped by polymerase chain reaction (PCR) using a couple of primers, namely, TAF: 5′-TCGTCCTTCTTCCTCTCTGG-3′ and TAR: 5′-TCCTGCTCCTGCCAGAGGTT-3′. Polymerase chain reaction was performed in 25 **μ*L* reaction volumes containing 100 ng of genomic DNA, 0.2 mmol/L of each dNTP, 50 mmol/L KCl, 15 mmol/L Tris-HCl PH 8.0, 2.5 mmol/L MgCl_2_, 0.5 U AmpliTaq polymerase (Invitrogen Life Technologies, Carlsbad, CA, USA), and 10 pmol of each forward and reverse primers. The PCR cycling conditions included an initial denaturation of 10 min at 96°C followed by 35 cycles of 96°C for 30 s, annealing at 58°C for 30 s, and extension at 72°C for 1 min. The run was ended by a final extension at 72°C for 7 min.

PCR products were then purified and doubly sequenced (forward and reverse) by ABI PRISM Big Dye Terminator on Ready Reaction Kit (Applied Biosystems, Foster City, CA, USA) and an ABI 310 DNA sequencer (PEApplied Biosystems, Foster City, USA).

### 2.3. Data Analysis

The sample of SCA patients was divided into two groups according to the presence or absence of cholelithiasis. 76 patients with normal hemoglobin (AA) and presented cholelithiasis were enrolled in the analysis. We compared demographic and hematological and clinical data between the groups of patients. As for *A(TA)*_n_ *TAA* polymorphism genetic differences between the groups were evaluated. We defined two intervals of total bilirubin levels. The first includes total bilirubin value <35 *μ*mol/L which is the critical value of total bilirubin associated with the Gilbert syndrome. The second interval includes bilirubin value higher than the cutting point 35 *μ*mol/L. We investigated the relationships between genotypes found and these intervals.

### 2.4. Statistical Analysis

The demographic and hematologic data are normally distributed, so we used means and standard deviations. The bilirubin data are not normally distributed, so we used medians. For each variable (demographic, hematological, and biochemical) differences between cases and controls were evaluated applying the *t*-test or the nonparametric Mann-Whitney test as appropriate using SPSS (version 18). The Hardy-Weinberg equilibrium was tested using the software package Arlequin (version 3.01). Genetic differences between cases and controls were evaluated applying exact tests to genotypic or allelic contingency tables using compare 2 (version 1.02). The relationships between genotypes found and total bilirubin level were evaluated applying Fisher's exact test using compare 2 (version 1.02). We calculated *P* values for the entire tests and Fisher's exact test and chi-squared test were used as appropriate. 

## 3. Results

### 3.1. Demographic, Hematological, and Biochemical Analysis

The distribution of each continuous variable was performed using the nonparametric Mann-Whitney test. Our results show that there is no significant difference between the two groups of SCA patient according to the presence or the absence of cholelithiasis (*P* > 0.05), whereas, the comparison of total conjugated and unconjugated bilirubin concentrations between the two groups of SS children patients shows a significant difference with *P* < 0.05. Our findings show a significant difference between SCA patients and patients with cholelithiasis considered as control group with *P* < 0.05 (Table 1).

### 3.2. Analysis of the *A(TA)*_n_ *TAA* Polymorphism

All samples were found to be in Hardy-Weinberg equilibrium (*P* = 0.09) for *A(TA)*_n_ *TAA* polymorphism. Our results show the presence of seven genotypes, namely, (TA)_5_/(TA)_6_, (TA)_6_/(TA)_6_, (TA)_6_/(TA)_7_, (TA)_7_/(TA)_7_, (TA)_5_/(TA)_7_, (TA)_7_/(TA)_8_, and (TA)_8_/(TA)_8_. The distribution of genotypes between children with gallstones and who are without gallstones and the control group are shown in Table 2. The comparison of these genotypes according to the number of (TA) reported that the genotypes (TA)_7_/(TA)_7_ and (TA)_7_/(TA)_8_ were significantly associated with SCA patients with gallstones (*P* < 0.05). Our results show a significant association between genotype (TA)_7_/(TA)_7_ and gallstones in the control group. Moreover, (TA)_7_ and (TA)_8_ allelic variants are found to be associated with gallstones in SCA children patients (*P* < 0.05) Table 2. Association is not found in the control group. 

### 3.3. Relationship between Total Bilirubin Levels and *A(TA)*_n_ *TAA* Polymorphism

The repartition of different genotypes depending on two intervals representing bilirubin level in SCA children shows that genotypes carrying (TA)_7_ and (TA)_8_ alleles are associated with increased bilirubin level. Similar results were found in the control group (Tables 3(a) and 3(b)).

## 4. Discussion

In the current study we tested 102 SCA Tunisian children patients among whom 52 have cholelithiasis and 76 subjects were chosen as control group characterized with normal hemoglobin status and presence of cholelithiasis and analyzed the polymorphism at the promoter and the relationships between the various *UGT1A1* promoter genotypes and alleles and bilirubin levels. In a previous study we were interested to determine the frequency of *A(TA)*_n_ *TAA* and Gly71Arg of *UGT1A1 *in a healthy population [21]. The polymorphism *A(TA)*_n_ *TAA* showed that genotype (TA)_7_/(TA)_7_ described as being associated with Gilbert's syndrome was encountered in 11% of the population studied. This percentage is close to the value described in the Caucasian population, estimated at 10% [22, 23]. Concerning the polymorphism Gly71Arg, our results show that the mutated allele is encountered in 15.7% of our studied population. This frequency differs greatly from that reported for Caucasians and Afro-Americans but it is similar to that perceived at the Japanese population [24–28]. All these results suggest that the Tunisian population appears to be heterogeneous for *UGT1A1* gene mutation status. The heterogeneity of Tunisian population for SCA haplotypes has been reported previously by Imen et al., 2011 [4]. The authors have demonstrated the predominance of the Benin haplotype in SCA patients suggests that the *β*S mutation present in Tunisia may have originated from the Benin region and was brought to Tunisia along the slave trade routes. However, they have reported the presence of another atypical haplotype that could be considered as specific to Tunisian chromosome *β*S. In a previous study we have been interested in the implication of the polymorphism *A(TA)*_n_ *TAA* of *UGT1A1* in occurrence of cholelithiasis among Tunisian patients with normal hemoglobin status. Our findings have showed that subjects with (TA)_7_ or (TA)_8_ variant in their genotypes are associated with high bilirubin level. Furthermore, we have demonstrated that (TA)_6_/(TA)_7_ and (TA)_7_/(TA)_7_ genotypes and (TA)_7_ and (TA)_8_ alleles were significantly associated with an increased risk of gallstone diseases *P* = 0.0017, *P* = 6.1 × 10^−6^,  *P* = 1.5 × 10^−6^, and *P* = 0.025, respectively [29]. In this study, our results show that total bilirubin level increased with the genotypes (TA)_6_/(TA)_7_, (TA)_7_/(TA)_7_, and (TA)_7_/(TA)_8_ (*P* = 7.1 × 10^−7^;  *P* = 2.4 × 10^−16^; and *P* = 8.2 × 10^−7^), respectively. In fact, the addition of an extra (TA) at the TATA box seems to interfere with binding of the transcription factor IID which is responsible for the transcription of *UGT1A1* gene. This interference leads to the reduced expression of *UGT1A1* and hence in the expression of bilirubin-UGT1 (30% of normal) [2]. Furthermore, our findings show a significant association between genotypes (TA)_7_/(TA)_7_ and (TA)_7_/(TA)_8_ and cholelithiasis in SCA children patients with a *P* value of 8.1 × 10^−8^ and *P* = 0.01, respectively. (TA)_7_ and (TA)_8_ allele variants are found to be associated with cholelithiasis among SCA children with *P* = 5.8 × 10^−9^ and *P* = 0.01, respectively. In order to interpret the effect of the *A(TA)*_n_ *TAA* promoter polymorphism on the lithogenesis in SCA patients our results were compared against those of a control group. We demonstrated that the genotype (TA)_7_/(TA)_7_ was associated with cholelithiasis in SCA and in the control group by cons (TA)_7_/(TA)_8_ was associated only among SCA children patients. Our results are similar with those of Chaar et al. on Guadeloupe SCA patients [7], where they demonstrated that frequency of cholelithiasis was significantly higher in both adult and children patients with (TA)_7_/(TA)_7_ and (TA)_7_/(TA)_8_ genotypes compared to those with other genotypes. Our results are similar to those of previous studies concerning (TA)_7_ variant which presents an excess risk for gallstone occurring in children patients with SCA and thalassemia (minor, intermedia, and *β* ^0^) [4–16]. Outside of hemolytic disease, (TA)_7_ variant has been reported by many studies to be associated with both hyperbilirubinemia and cholelithiasis [25, 26, 28, 30, 31]. Herein, we demonstrated the association of the genotype (TA)_7_/(TA)_7_ with cholelithiasis in SCA patients and in the control group. Our study is the first findings about the implication of *A(TA)*_n_ *TAA* of *UGT1A1* in lithogenesis among SCA children patients in Tunisia. Our data confirmed the role of (TA)_7_ variant and highlighted the role of (TA)_8_ in early gallstones formation; association is not found in previous studies. As future directions of our research, we will focus on the Gly71Arg polymorphism in the first exon of the *UGT1A1* gene reported to be associated with the same phenotypes [2, 25, 27, 32, 33]. Also we will focus on other candidate genes which can be associated with both hyperbilirubinemia and cholelithiasis in SCA such as SLCO1BI and SLCO1A2 [34].

## 5. Conclusion

The novelty of this report is that it is the first time that a similar study was made on the Tunisian children sickle cell population and that the results show a clear association of (TA)_7_/(TA)_7_ and (TA)_7_/(TA)_8_ genotypes as well as the (TA)_7_ allele with the cholelithiasis and hyperbilirubinemia. Interestingly our findings show the association of (TA)_8_ allele with the cholelithiasis, association not described previously in other population.

## Figures and Tables

**Table 1:** Hematological, demographic, and clinical data of studied population.

	Normal values		SCA patients with cholelithiasis	SCA patients without cholelithiasis	Patients with cholelithiasis	*P*1	*P*2
	M	F					
Number			52 SS	50 SS	76 AA		
Age (mean)			13 ± 2.9	10 ± 3.6	35 ± 5	0.425	0.020
Sex ratio (M/F)			24/28	20/30	33/43	0.423	0.323
Hb (g/dL)	13–18	12–16	7.3 ± 0.9	7.9 ± 1.3	13 ± 0.15	0.521	0.035
RBC (1012/L)	4.5–6.2	4–5.4	2.89 ± 1.02	3.29 ± 0.9	4.80 ± 0.7	0.270	0.120
MCV (fL)	80–100	80–100	77.2 ± 1.3	79.7 ± 0.9	85 ± 2	0.560	0.049
MCH (pg)	27–32	27–32	35.7 ± 1.02	34.9 ± 2.1	30.2 ± 1.03	0.100	0.130
RDW (%)	11–14	11–14	5.29 ± 1.02	4.83 ± 0.5	13.2 ± 0.2	0.579	0.029
HbA (%)	97-98	97-98	0	0	97 ± 0.2	1	0.012
HbS (%)	0	0	86.4 ± 0.4	86 ± 0.3	0	1	0.012
HbF (%)	0	0	10.6 ± 0.3	11 ± 0.1	0	1	0.012
HbA2 (%)	2-3	2-3	3 ± 0.1	3 ± 0.2	3 ± 0.2	1	0.012
Total bilirubin level (*μ*mol/L)	<17	<17	80.25	53.5	30.3	0.001	0.020
Unconjugated bilirubin level (*μ*mol/L)	<14	<14	70.12	38.2	25.8	0.001	0.035
Conjugated bilirubin level (*μ*mol/L)	<14	<14	10.13	15.3	4.5	0.001	0.120

Usual value of total bilirubin level is <17 *μ*mol/L.

SS: homozygous of *β*-globin gene mutation.

AA: normal adult hemoglobin.

The demographic and hematologic values are indicated as mean ± standard deviation.

The bilirubin values are indicated as medians.

Hb: hemoglobin, RBC: red blood cell, MCV: mean corpuscular volume, MCH: mean corpuscular hemoglobin, and RDW: red blood distribution width.

Statistics for the comparison of demographic and hematological variables between the two groups were performed using the *t*-test and chi-square test as appropriate (SPSS 18.0).

Statistics for the comparison of bilirubin level between the two groups were performed using the nonparametric Mann-Whitney test (SPSS 18.0).

*P*: index of significance, each *P* < 0.05 is considered as significant. *P*1: comparison between SCA patients according to the presence or the absence of cholelithiasis. *P*2: comparison between SCA patients without cholelithiasis and patients with cholelithiasis.

**Table 2:** Distribution of *A(TA)_n_TAA* genotypes and allele frequency according to the presence or absence of cholelithiasis in the sample of patients.

	Children without cholelithiasis *N* = 50	Children with cholelithiasis *N* = 52	Control group *N* = 76	*P*1	*P*2
Genotypes					
(TA)_6_/(TA)_6_	30	10	30	1*	1*
(TA)_6_/(TA)_5_	1	1	0	0.46	1
(TA)_6_/(TA)_7_	16	15	31	0.07	0.145
(TA)_7_/(TA)_7_	1	20	11	8.1 × 10^−8^	9.5 × 10^−3^
(TA)_5_/(TA)_7_	1	1	1	0.46	1
(TA)_7_/(TA)_8_	1	5	2	0.01	1
(TA)_8_/(TA)_8_	0	0	1	1	1
Allele frequency					
(TA)_6_	0.77	0.346	0.598	1*	1*
(TA)_5_	0.02	0.019	0.0065	0.59	0.433
(TA)_7_	0.20	0.586	0.368	5.8 × 10^−9^	0.310
(TA)_8_	0.01	0.048	0.025	0.01	0.664

1*: reference group.

*P*: index of significance with Yates's correction, each *P* < 0.05 is considered as significant.

Control group: patients with normal hemoglobin status and presented cholelithiasis.

*P*1: comparison between SCA patients according to the presence or the absence of cholelithiasis, *P*2: comparison between SCA patients without cholelithiasis and the control group.

**Table:** (a)

*A(TA)_n_TAA* genotypes	*A*	*B*	*P*
(TA)_6_/(TA)_5_	2	0	—
(TA)_5_/(TA)_7_	2	0	—
(TA)_6_/(TA)_6_	40	0	1*
(TA)_6_/(TA)_7_	18	15	7.1 × 10^−7^
(TA)_7_/(TA)_7_	0	20	2.4 × 10^−16^
(TA)_7_/(TA)_8 _	0	5	8.2 × 10^−7^

Total	62	40	

**Table:** (b)

*A(TA)_n_TAA * genotypes	*A*	*B*	*P*
(TA)_6_/(TA)_5_	0	0	—
(TA)_5_/(TA)_7_	1	0	—
(TA)_6_/(TA)_6_	30	0	1*
(TA)_6_/(TA)_7_	0	31	5.6 × 10^−10^
(TA)_7_/(TA)_7_	0	11	3.2 × 10^−10^
(TA)_7_/(TA)_8_	0	2	2 × 10^−3^
(TA)_8_/(TA)_8_	0	1	0.119

Total	31	45	

*A*: total bilirubin level comprising between 15 and 34 *μ*mol/L.

*B*: total bilirubin level comprising between 35 and 100 *μ*mol/L.

Usual value of bilirubin level: total bilirubin <17 *μ*mol/L.

1*: reference group.

*P*: index of significance, each *P* < 0.05 is considered as significant.

## Abbreviations

CI: Confidence interval
OR: Odds ratio
*P*: Index of significance
SCA: Sickle cell anemia
*UGT1A1*: Uridine diphosphoglucuronosyltransferase 1A1.
